# Effects of slight shading in summer on the leaf senescence and endogenous hormone and polyamine contents in herbaceous peony

**DOI:** 10.1038/s41598-023-46192-y

**Published:** 2023-10-31

**Authors:** Anqi Xie, Mengwen Lv, Dongliang Zhang, Yajie Shi, Lijin Yang, Xiao Yang, Jie Du, Limin Sun, Xia Sun

**Affiliations:** 1 College of Horticulture, Shandong Agricultural University, State Key Laboratory of Crop Biology, Tai’an, 271018 Shandong China; 2 College of Forestry, Shandong Agricultural University, Tai’an, 271018 Shandong China; 3 School of Bioengineering, Huainan Normal Unversity, Huainan, 232038 Anhui China; 4 College of Landscape Architecture, Beijing Forestry University, Beijing, 100083 China

**Keywords:** Biological techniques, Physiology, Plant sciences

## Abstract

Herbaceous peony is a perennial root plant that likes light and is cold-resistant. During summer, high temperature and strong light intensity advance its entry into the leaf wilting stage, which limits the accumulation of nutrients and formation of strong buds and severely affects its growth and development the following year. In this study, the wild herbaceous peony species and two main cultivars, ‘Zifengyu’ and ‘Hongfengyu’, were subjected to slight shading and strong light environments in summer, and their effects on leaf senescence and endogenous hormone and polyamine contents were explored. Slight shading treatment significantly delayed withering, increased the leaf net photosynthetic rate, and increased the chlorophyll, soluble sugar, indole-3-acetic acid, zeatin, gibberellin, spermine, spermidine, putrescine, and polyamine contents. Additionally, slight shading significantly reduced the proline and abscisic acid contents. Slight shading during summer prolonged the green period and delayed leaf senescence. The tolerance of tested materials to strong light intensity in summer was ranked as follows: ‘Zifengyu’ > ‘Hongfengyu’ > wild species. In conclusion, this study revealed that summer leaf senescence is delayed in herbaceous peony through shading and growth regulators. Additional varieties should be evaluated to provide reference for high-efficiency, high-quality, and high-yield cultivation of herbaceous peony.

## Introduction

Light is a crucial environmental factor affecting plant growth. Specifically, plant height, canopy width, and leaf morphology vary widely under different light conditions^1^. Herbaceous peony (*Paeonia lactiflora* Pall.) is a temperate, long-day perennial herb in the Paeoniaceae family. High temperature and light intensity during summer can result in leaf scorch, early plant chlorosis, and premature withering; this time is the key period of nutrient storage in herbaceous peony, which severely affects its nutrient accumulation, strong bud formation, and growth in the following year^2^.

Chlorophyll and protein degradation and a decrease in the photosynthetic rate occur during plant senescence. Plant senescence is closely related to regulating endogenous hormones and polyamines^3^. Such as auxins, gibberellins, and cytokinins, which are considered to be anti-aging hormones^4–6^. In contrast, abscisic acid (ABA) positively impacts leaf senescence^7^. Hormones are superior messengers of polyamines and can affect the metabolism of tissue polyamines by influencing polyamine synthesis, precursors, or other pathways. Similarly, polyamines affect hormone levels through various pathways^8^. Polyamines are involved in key developmental and physiological metabolic processes such as plant cell growth, stress resistance, senescence, and maturation^9,10^. Multiple studies have shown that exogenous application of polyamines or an increased content of endogenous polyamine can delay aging^11–14^.

As a simple and inexpensive technology, shading can prevent summer leaf scorching, allowing plants to perform photosynthesis and delaying plant aging^15^. Zhao et al.^16^ found that shading alleviated heat damage in herbaceous peony under high-temperature stress by removing reactive oxygen species, protecting cell structures, enhancing photosynthesis, and increasing the expression levels of heat shock proteins. Similarly, Du et al. (2018) ^2^ found that summer shading reduced the light intensity, thereby reducing the degree of photo-inhibition of photosystem II in herbaceous peony leaves. In this case, the photoprotection mechanism of the lutein cycle pathway was active; however, fluorescence, such as non-photochemical chlorophyll a quenching, was low.

Many hypotheses have been proposed to explain plant leaf senescence; the most prominent hypotheses involve hormone balance, calcium regulation, and membrane lipid peroxidation ^17^. However, the effects of summer shading on endogenous hormone and polyamine contents in herbaceous peony leaves have not been reported. In this study, wild species of herbaceous peony and the ‘Zifengyu’ and ‘Hongfengyu’ varieties at different leaf wilting periods were evaluated to explore the effects of summer slighting shading on the changes in leaf endogenous hormones and polyamines in herbaceous peony and to analyze the regulation mechanism of endogenous hormones and polyamines on leaf senescence. This study provides a theoretical basis for the application of summer shading facilities and growth regulators.

## Materials and methods

### Tested materials and methods

The main cultivars of *Paeonia lactiflora* Pall (namely ‘Zifengyu’ and ‘Hongfengyu’) and the wild species used in this study were collected from the Herbaceous Peony Resource Nursery and Horticultural Experimental Center of Shandong Agricultural University (35°38′–36°28′ N, 116°20′–117°59′ E), and the plant and seedling collection permission was obtained. Experimental research on plants in this study, including the collection of plant materials, complied with relevant institutional, national, and international guidelines and legislation.

The study was conducted from June 2021 to September 2021 at the Herbaceous Peony Resource Nursery and Horticultural Experimental Center of Shandong Agricultural University, in Tai'an City, Shandong Province, China. The test station is located in a temperate semi-humid continental monsoon climate area with four distinct seasons and rain and heat occurring in the same season. Spring and autumn are short, whereas winter and summer are long, with an annual average temperature of 11–14 °C, extreme low temperature of − 20 °C, extreme high temperature of 41 °C, frost-free period of about 187 days, average annual precipitation of 600–700 mm, and relative annual humidity of 65%. The temperature during the test period was 14–36 °C. Light intensity was measured using a TES-1339 illuminance meter (Zhuhai Tianchuang Instrument Co., Ltd., Zhuhai, China). The maximum light intensity can reach 89,650 Lx under natural strong light and 62,755 Lx under slight shading.

**Figure 1:**
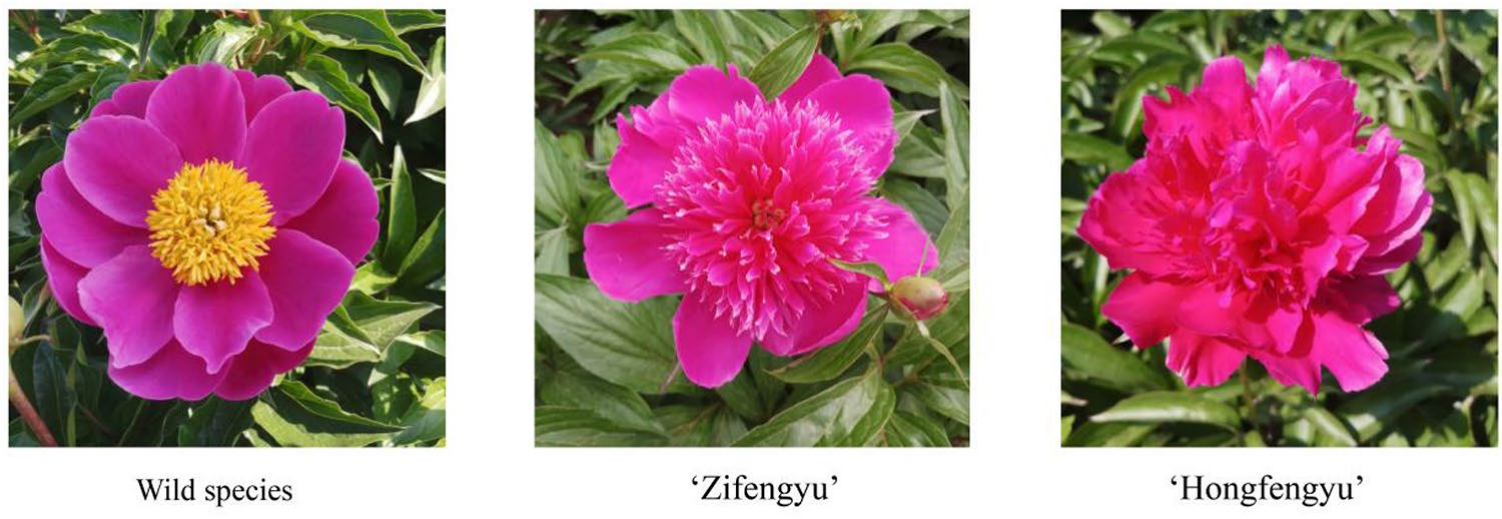
Herbaceous peony species and cultivars used in the study.

Wild species of herbaceous peony and the main cultivars ‘Zifengyu’ and ‘Hongfengyu’, which entered the dead leaf stages at different times, were selected as the tested materials (Fig. 1). Three-year seedlings showing healthy and consistent growth and without disease and insect pests were selected. A spacing of 80 × 70 cm between plants and conventional management were applied. The tested materials were treated with natural strong light (SL) or slight shading (SS) between June 20, 2021 and the onset of leaf wilting. The leaf wilting rate was determined as the number of non-green (purple red, yellow) leaves divided by the number of total leaves; when the rate reached ≥ 80%, the plants were considered to be in the leaf wilting stage. Plants with values < 80% were considered to be in the green stage. There were three replicates for each treatment, and each replicate contained 20 strains. Based on preliminary results, the shading material was selected as a 3-pin sunshade net commonly used in production, and the height above the ground was set at about 1.8 m. By adjusting the height of the net, the shading rate was kept stable at 30–35%.

Starting on June 20, 2021, the treated leaves were sampled at 08:00 h every 10 days, transported to the laboratory after quick-freezing in liquid nitrogen, and stored at − 80 °C until further use. The sampling cut-off date was when leaf wilting rate of ≥ 80%.

### Determination of leaf net photosynthetic rate and photosynthetic characteristics

Based on the method described in Du^2^, the CIRAS-3 (PP Systems, USA) portable photosynthesis analyzer was used to determine the leaf net photosynthetic rate, diurnal changes in light quantum flux density (PAR), leaf temperature (T1), air temperature (Ta), water use efficiency (WUE), and air CO_2_ concentration. Five representative leaves were selected for each test variety, and the determination was repeated three times. The net photosynthetic rate was measured from 09:00 to 10:30 h. It should be noted that the external environment for measuring the net photosynthetic rate had to be sunny and breezy.

### Determination of leaf microstructure

Based on the method described in Du^2^, the leaves were cut into 5 cm × 5 cm pieces and immediately placed in a Formalin-Aceto-Alcohol (FAA) fixing solution, rinsed after removal, completely dehydrated with ethanol, and soaked in a mixed solution of ethanol and xylene. They were then sliced with a xylene transparent, wax-through and embedded, and dyed with an afrutin reagent, after which excess reagent was removed and neutral resin was added. Sections were photographed with a three-lens photographic microscope (Motic-BA400, McOddy Industrial Group Co., LTD., Xiamen, China) and measured with the Image-Pro Plus 6.0 software.

### Determination of physiological indicators

Based on the method described by Cang and Zhao^18^, the chlorophyll content was determined using 80% ethanol extraction followed by colorimetry. The net photosynthetic rate was measured using a CIRAS-3 portable photosynthetic apparatus (PP Systems, Amesbury, MA, USA), and the soluble protein and proline contents were determined via anthrone colorimetry and sulfosalicylic acid method, respectively.

### Determination of endogenous hormone contents

The indole-3-acetic acid (IAA), zeatin (ZR), gibberellic acid (GA_3_), and ABA contents were estimated via high-performance liquid chromatography (HPLC) (Waters 2487, Waters Corp., Milford, MA, USA) using a Novapak C18 column (250 × 4.6 mm, 5 µm; Sigma Aldrich, St. Louis, MO, USA), following the methodology of Zhang et al.^19^. The mobile phase was a mixture of methanol and 1% acetic acid at a ratio of 44:56. The wavelength was 254 nm and column temperature was 30 °C. The sample size was 15 μL and flow rate was 0.8 mL min^−1^.

### Determination of endogenous polyamine (PA) contents

According to Liu et al.^20^, spermidine (Spd), putrescine (Put), spermine (Spm), and PAs were determined using HPLC as described in Section "Determination of leaf microstructure". The mobile phase comprised methanol and water; the detection wavelength was 230 nm, flow rate was 1 mL·min^-1^, and column temperature was 30 °C.

### Data analysis

The SPSS Statistics software version 22.0 (SPSS, Inc., Chicago, IL, USA) was used for the minimum significant difference test, and Duncan’s new multiple range test was used to compare the SL and SS treatment samples. The Microsoft Excel software (Microsoft, Redmond, WA, USA) was used for the analysis and creation of graphs. All biological measurements were repeated three times.

## Results

### Changes in leaf photosynthetic characteristics under strong light and slight shading treatments

**Figure 2:**
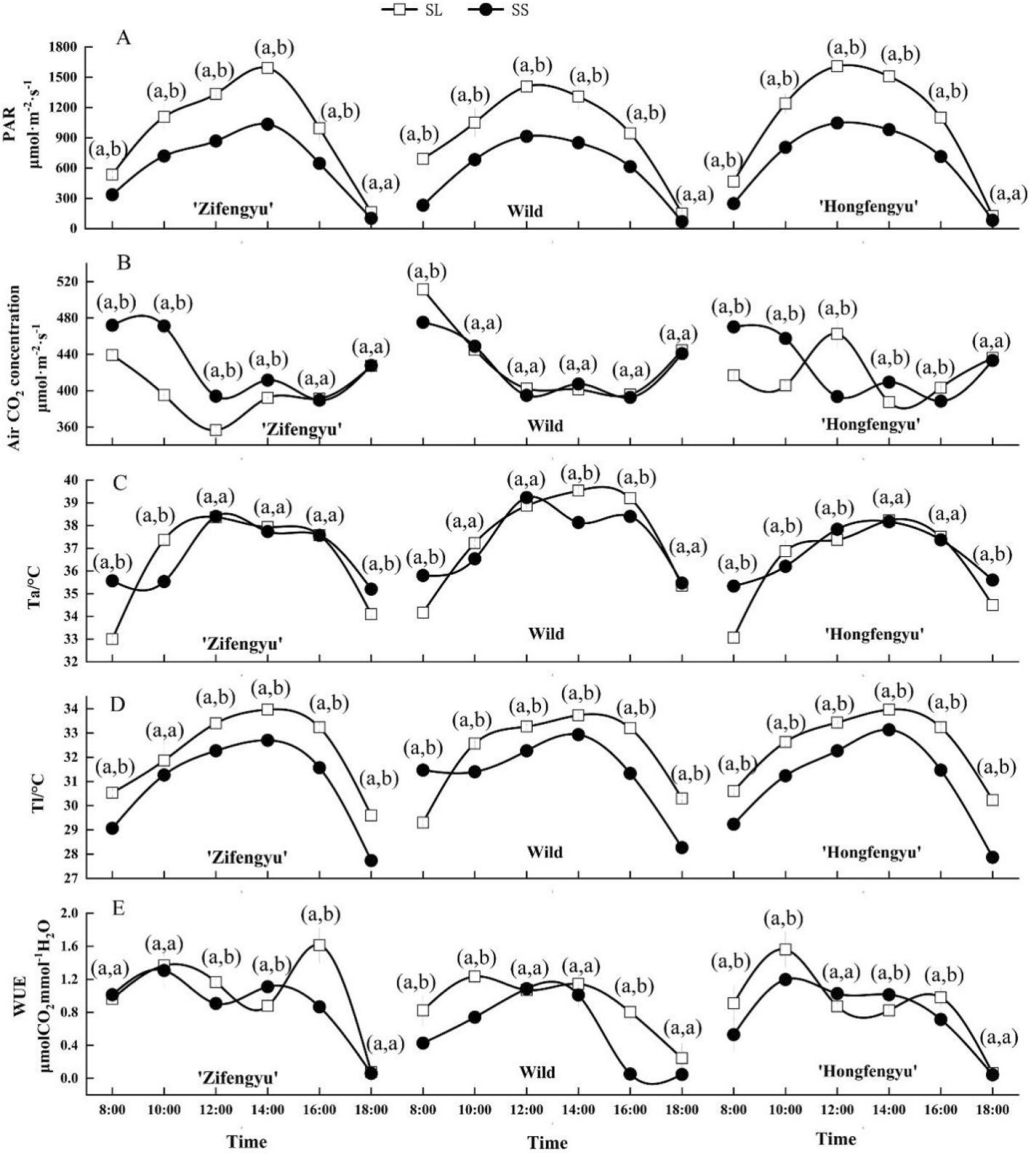
Changes in the diurnal variation of PAR, air CO_2_ concentration, Ta, Tl, and WUE under the SL and SS treatments. SL, strong light; SS, slight shading. SL, strong light; SS, slight shading. Data are the mean ± standard error (*n* = 3). Different letters indicate significant differences among treatments according to Duncan’s test (*P* < 0.05).

The diurnal variation curves of the optical quantum flux density (PAR), leaf temperature (T1), and air temperature (Ta) of the tested materials under SL and SS treatments showed a unipeak trend, with the peak values in the period of 12:00–14:00 (Fig. 2). The SS treatment significantly reduced the PAR and T1 of the tested materials. The diurnal variation curve of water use efficiency (WUE) in the SS treatment showed a unipeak trend, while that in the SL treatment showed a bimodal trend. The air CO_2_ concentration of ‘Hongfengyu’ in the SS treatment was significantly increased by 12.83% and 12.77% at 8:00 and 10:00, respectively, compared with that in the SL group, and significantly decreased by 14.92% at 12:00 compared with that in the SL treatment. Compared with that in the SL treatment, the air CO_2_ concentration of ‘Zifengyu’ in the SS treatment increased by 7.5%, 19.2%, 10.50%, and 4.98% during the period of 8:00–14:00, respectively. There was no significant difference in the air CO_2_ concentration of wild species between the SL and SS groups.

### Changes in leaf wilting rates under strong light and slight shading treatments

**Figure 3:**
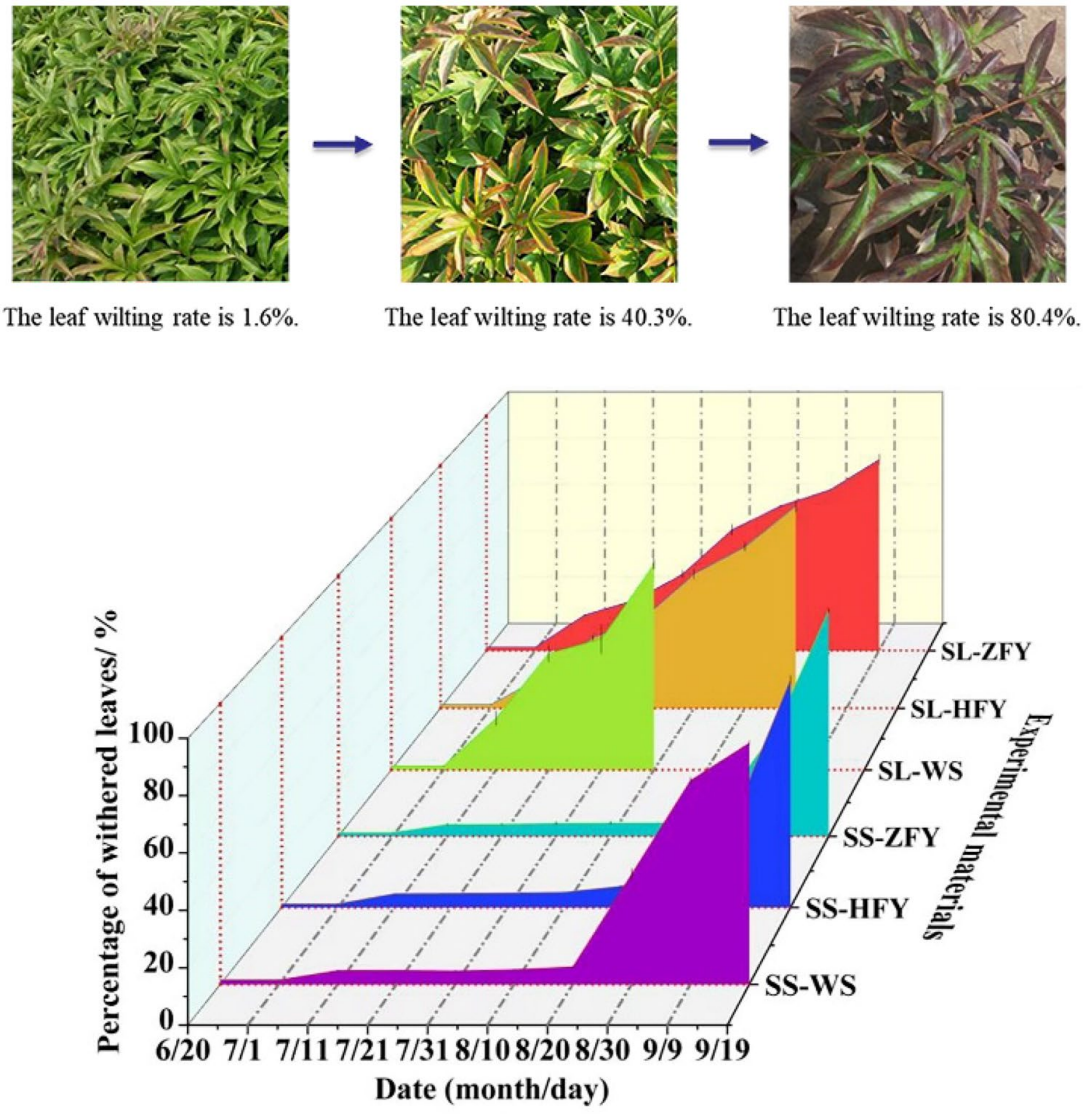
Changes in leaf wilting rates under the SL and SS treatments. SL, strong light; SS, slight shading; WS, wild species; ZFY, ‘Zifengyu’; HFY, ‘Hongfengyu’.

As shown in Fig. 3, the time required by the leaves of the tested materials in the SS group to reach an 80% leaf wilting rate was significantly longer than that required by those in the SL group. The tested materials in the SL group reached the natural leaf wilting period in the order of wild species (August 10) > ‘Hongfengyu’ (August 30) > ‘Zifengyu’ (September 9). Before August 20, the tested materials in the SS group maintained a low leaf wilting rate. The leaf wilting rate of wild species in the SS group rapidly increased after August 20, reaching a maximum of 85.7% on September 19. The leaf wilting rates of ‘Zifengyu’ and ‘Hongfengyu’ in the SS group remained low before August 30 and reached a maximum on September 19 with values of 85% and 83.1%, respectively. September 19 was the common leaf wilting period of all tested materials in the SS group. At this time, the climate temperature was no longer suitable for the growth of herbaceous peony, and the aboveground part gradually entered the dormant period. Slight shading prolonged the green stage of ‘Zifengyu’, ‘Hongfengyu,’ and wild species for 10, 20, and 40 days, respectively. Different genotypes of herbaceous peony showed different ranges of adaptation to light intensity, and the light intensity tolerated by the tested materials in this experiment was in the order of ‘Zifengyu’ > ‘Hongfengyu’ > wild species.

### Changes in leaf microanatomical characteristics under strong light and slight shading treatments

**Figure 4:**
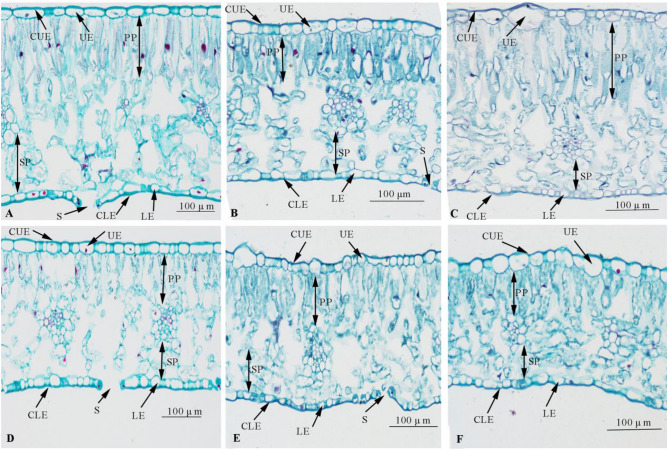
Changes in leaf microanatomical characteristics under the SL and SS treatments. SL, strong light; SS, slight shading. (**A**) SL treatment ‘Zifengyu’; (**B**) SL treatment ‘Hongfengyu’; (**C**) SL treatment wild species; (**D**) SS treatment ‘Zifengyu’; (**E**) SS treatment ‘Hongfengyu’; (**F**) SS treatment wild species; CLE, cuticle of lower epidermis; S, stomata; LE, lower epidermis; CUE, cuticle of the upper epidermis; UE, upper epidermis; PP, palisade tissue; SP, spongy tissue.

As shown in Fig. 4 and Table 1, the leaf thickness of the tested materials under the SL treatment was significantly higher than that under the SS treatment. Herbaceous peony maintained normal growth by increasing leaf thickness to block direct sunlight. The upper epidermal thickness of ‘Hongfengyu’ and wild species under the SL treatment was significantly higher than that under the SS treatment. There was no significant difference in the upper epidermal thickness of ‘Zifengyu’ between the SL and SS treatments. The lower epidermis thickness of the tested materials under the SS treatment was significantly higher than that under the SS treatment. This is important because the thickening of the upper and lower epidermis is conducive to reducing the direct influence of strong light on the leaves in summer.

**Table 1:** Comparison of leaf epidermal structures of the tested materials under the SL and SS treatments.

Index (μm)	‘Zifengyu’		‘Hongfengyu’		Wild species	
	SS treatment	SL treatment	SS treatment	SL treatment	SS treatment	SL treatment
Leaf thickness	316.1 ± 60.7c	359.8 ± 16.9b	197.6 ± 15.9e	269.0 ± 14d	191.2 ± 62.3e	399.7 ± 18.1a
Upper epidermal thickness	22.8 ± 3.9b	22.0 ± 3.2b	17.2 ± 3.0c	22.4 ± 4.4b	23.6 ± 4.6b	30.9 ± 8.2a
Lower epidermal thickness	22.1 ± 5.0a	23.3 ± 6.5a	15.1 ± 4.8d	19.3 ± 3.4b	17.1 ± 2.6c	21.9 ± 4.4a
Upper cuticle thickness	4.3 ± 1.0b	5.4 ± 0.4a	3.5 ± 4.9c	2.5 ± 0.4e	2.6 ± 0.644de	2.9 ± 0.5d
Lower cuticle thickness	4.1 ± 0.9c	5.7 ± 0.5a	2.7 ± 0.8d	2.1 ± 0.2e	2.2 ± 0.4e	5.0 ± 1.1b

Data are shown as mean ± standard error (*n* = 3). Different letters indicate significant differences among treatments ng treatments Duncan’s test (*P* < 0.05).

Tight palisade and loose spongy tissues can effectively reduce the burn of strong light so that the diffractive light source can be fully used for photosynthesis. The greater the thickness of palisade tissue and leaf tightness, the stronger the leaf’s stress resistance. As shown in Fig. 4 and Table 2, the leaf tightness of ‘Zifengyu’ and wild species under the SL treatment was significantly higher than that under the SS treatment, whereas there was no significant difference in the leaf tightness of ‘Hongfengyu’ between the SL and SS treatments. Spongy tissue is generally thought to be unrelated to stress resistance, but has a certain plasticity in response to the environment. The thickness of spongy tissue of the tested materials under the SL treatment was significantly higher than that under the SS treatment. The ratio of palisade tissue thickness to spongy tissue thickness of ‘Zifengyu’ under the SS treatment was significantly higher than that under the SL treatment. The ratio of palisade tissue to spongy tissue of the wild species under the SS treatment was significantly lower than that under the SL treatment. There was no significant difference in leaf porosity of the tested materials between the SS treatment and SL treatments.

**Table 2:** Comparison of mesophyll tissue structures of the tested materials under the SL and SS treatments.

Index (μm)	‘Zifengyu’		‘Hongfengyu’		Wild species	
	SS treatment	SL treatment	SS treatment	SL treatment	SS treatment	SL treatment
Mesophyll thickness	195.4 ± 4c	344.5 ± 7.6a	170.0 ± 10.5d	254.4 ± 4.5b	145.6 ± 5.5e	350.0 ± 13.2a
Palisade tissue thickness	112.7 ± 37c	131.5 ± 16b	62.1 ± 12.7e	88.9 ± 11.6d	59.0 ± 9.4e	193.5 ± 28.5a
Spongy tissue thickness	156.9 ± 58bc	194.2 ± 13.4a	111.9 ± 20.1d	149.5 ± 12.8c	85.7 ± 14.2e	164.6 ± 23.9b
Palisade cell width	19.2 ± 5.2e	24.7 ± 6.1bc	22.4 ± 5.9d	23.1 ± 3.4 cd	25.4 ± 5.6b	28.4 ± 8.5a
Palisade cell length	57.2 ± 10.8d	101.3 ± 14.3b	60.8 ± 14.5d	90.6 ± 7.8c	62.0 ± 10.3d	143.7 ± 35.0a
Ratio of palisade tissue thickness to spongy tissue thickness	1.0 ± 0.9b	0.7 ± 0.1c	0.6 ± 0.2d	0.6 ± 0.1d	0.7 ± 0.20c	1.2 ± 0.3043a
Blade tightness	0.3 ± 0.065c	0.4 ± 0.036b	0.3 ± 0.07c	0.3 ± 0.04c	0.32 ± 0.078c	0.48 ± 0.061a
Leaf porosity	0.5 ± 0.165b	0.5 ± 0.039b	0.6 ± 0.068a	0.6 ± 0.04a	0.46 ± 0.102c	0.41 ± 0.0623c

Data are shown as mean ± standard error (n = 3). Different letters indicate significant differences among treatments according to Duncan’s test (*P* < 0.05).

### Changes in leaf chlorophyll content and net photosynthetic rate under strong light and slight shading treatments

**Figure 5:**
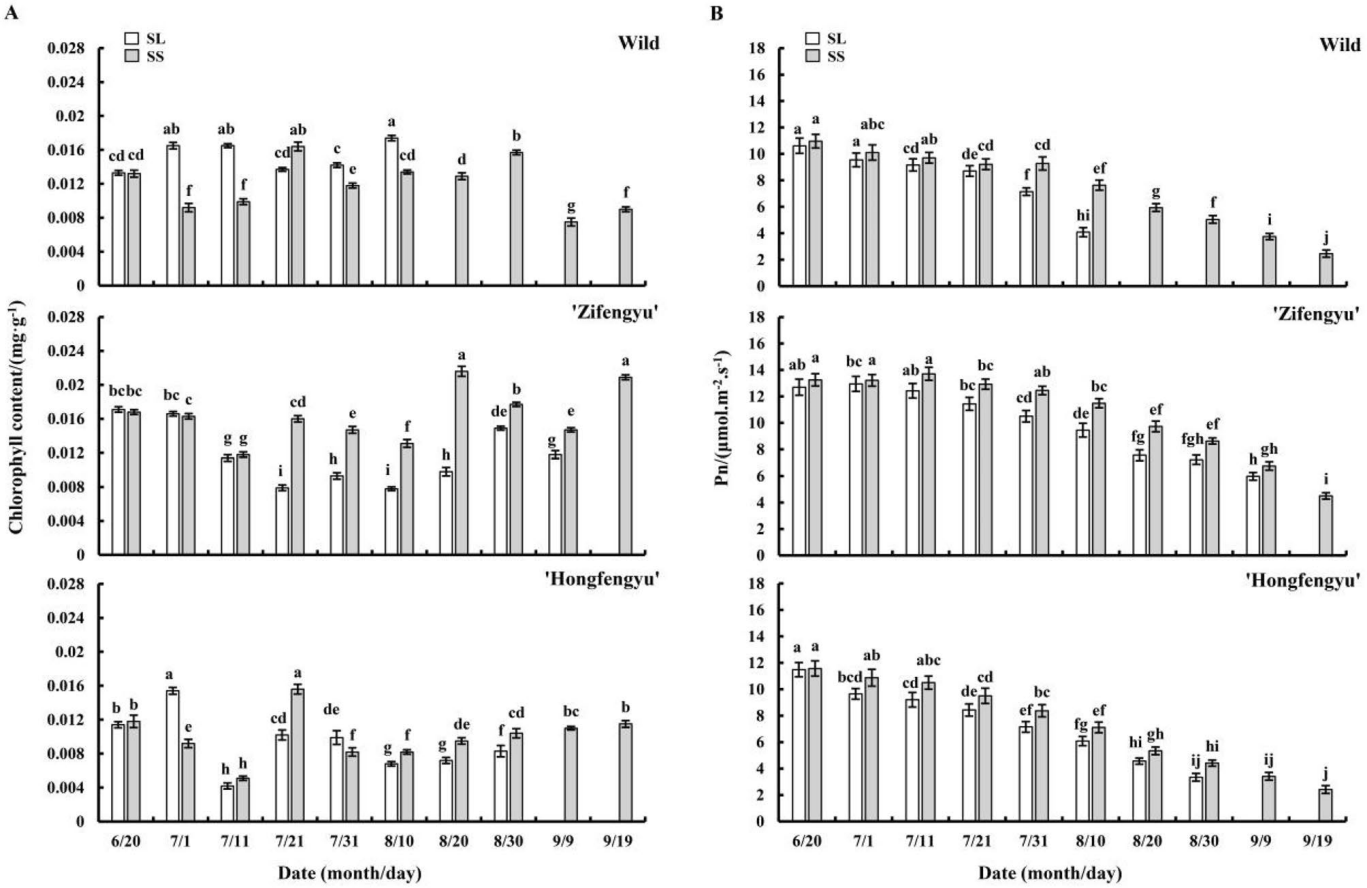
Changes in leaf chlorophyll content and net photosynthetic rate under the SL and SS treatments. SL, strong light; SS, slight shading. Data are shown as mean ± standard error (*n* = 3). Different letters indicate significant differences among treatments according to Duncan’s test (*P* < 0.05).

The SS treatment increased the leaf chlorophyll content of ‘Zifengyu’ and ‘Hongfengyu’; however, the opposite effect was observed in the wild species (Fig. 5A). From June 20 to September 19, under the SS and SL treatments, the leaf chlorophyll contents of ‘Zifengyu’ and ‘Hongfengyu’ showed an overall trend of first decreasing and then increasing, whereas the leaf chlorophyll content of the wild species showed a bimodal trend.

Over time, the net leaf photosynthetic rate of all tested materials under the SS and SL treatments showed a decreasing trend (Fig. 5B). The leaf net photosynthetic rate of all tested materials under the SS treatment was higher than that under the SL treatment. The results showed that in summer, the SS treatment increased the leaf net photosynthetic rate of herbaceous peony.

### Changes in leaf soluble protein and proline contents under strong light and slight shading treatments

**Figure 6:**
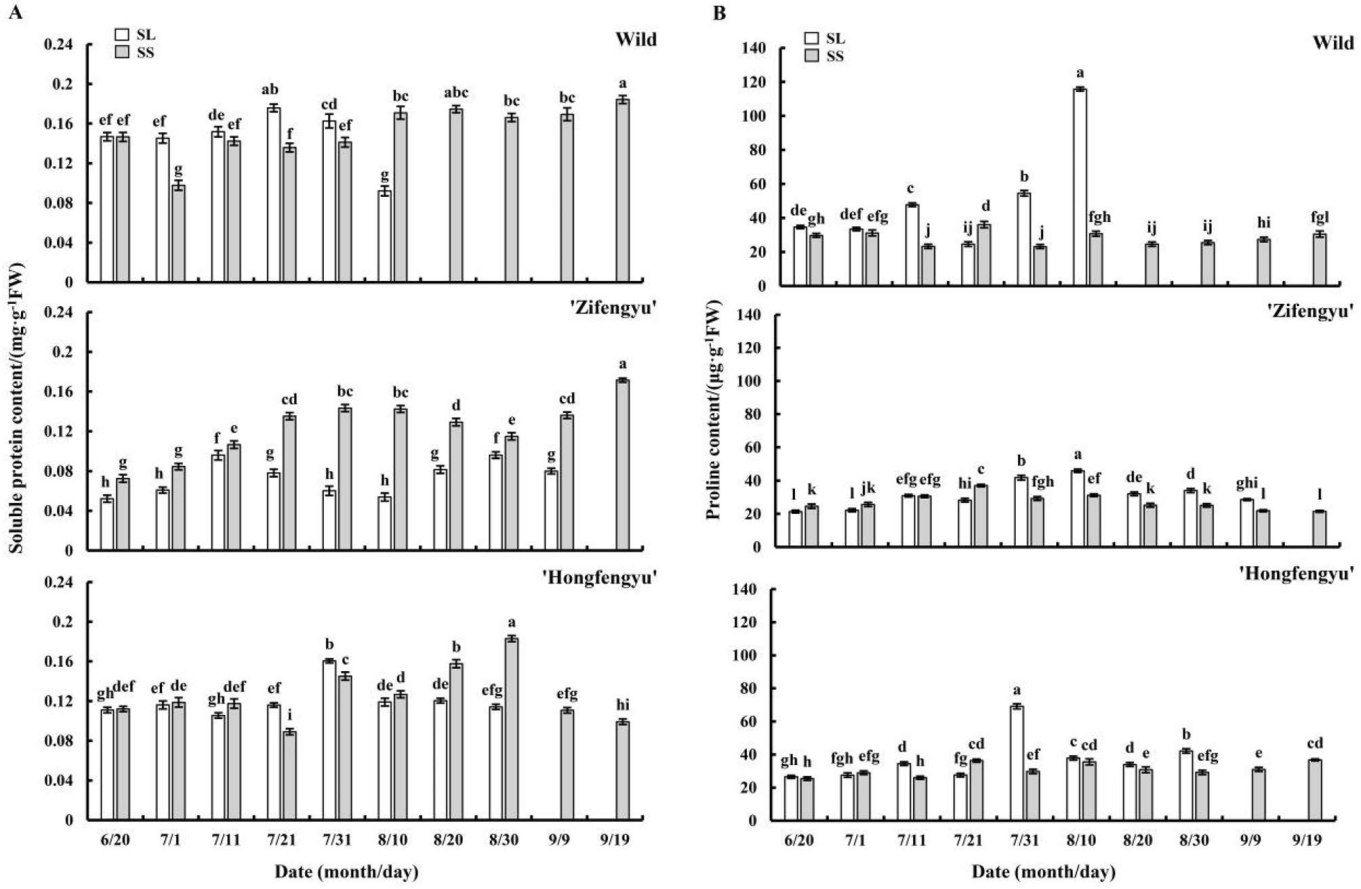
Changes in leaf soluble protein and proline contents under the SL and SS treatments. SL, strong light; SS, slight shading. Data are shown as mean ± standard error (*n* = 3). Different letters indicate significant differences among treatments according to Duncan’s test (*P* < 0.05).

As shown in Fig. 6A, the soluble protein content of ‘Zifengyu’ leaves under the SL and SS treatments showed a bimodal trend; the peak values were recorded on July 11 and August 30 under the SL treatment and on July 21 and September 19 under the SS treatment. The soluble protein content of ‘Zifengyu’ under the SS treatment was significantly higher than that under the SL treatment. The trend in the variation of the soluble protein content of ‘Hongfengyu’ under the SS and SL treatments was similar before August 10, and after August 20, that under the SS treatment was significantly higher than that under the SL treatment. The variation in the soluble protein content of wild species under the SL treatment showed a unipeak, reaching a peak on July 21. The soluble protein content of wild species under the SS treatment was lower than that under the SL treatment before July 31, whereas it was higher than that under the SL treatment on August 10 and remained high thereafter. The SS treatment increased the leaf soluble protein content of the tested materials; however, the effect was not evident in the early stage and even lower than SL treatment in the wild species, but it could significantly increase the soluble protein content in the later stage.

The SS treatment reduced the proline content of the leaves of the tested materials (Fig. 6B). From June 20 to September 19, the proline content of ‘Zifengyu’ leaves under the SS treatment showed a unipeak trend, peaking on July 21. The proline content in the leaves of ‘Hongfengyu’ and wild species under the SS treatment remained stable. From June 20 to September 19, the leaf proline contents of ‘Zifengyu’ and ‘Hongfengyu’ under the SL treatment showed a unipeak trend, showing peaks on August 10 and July 31, respectively. However, the leaf proline content of wild species under the SL treatment continued increasing and reached a maximum on August 10.

### Changes in leaf hormone levels under strong light and slight shading treatments

#### Changes in endogenous IAA content

**Figure 7:**
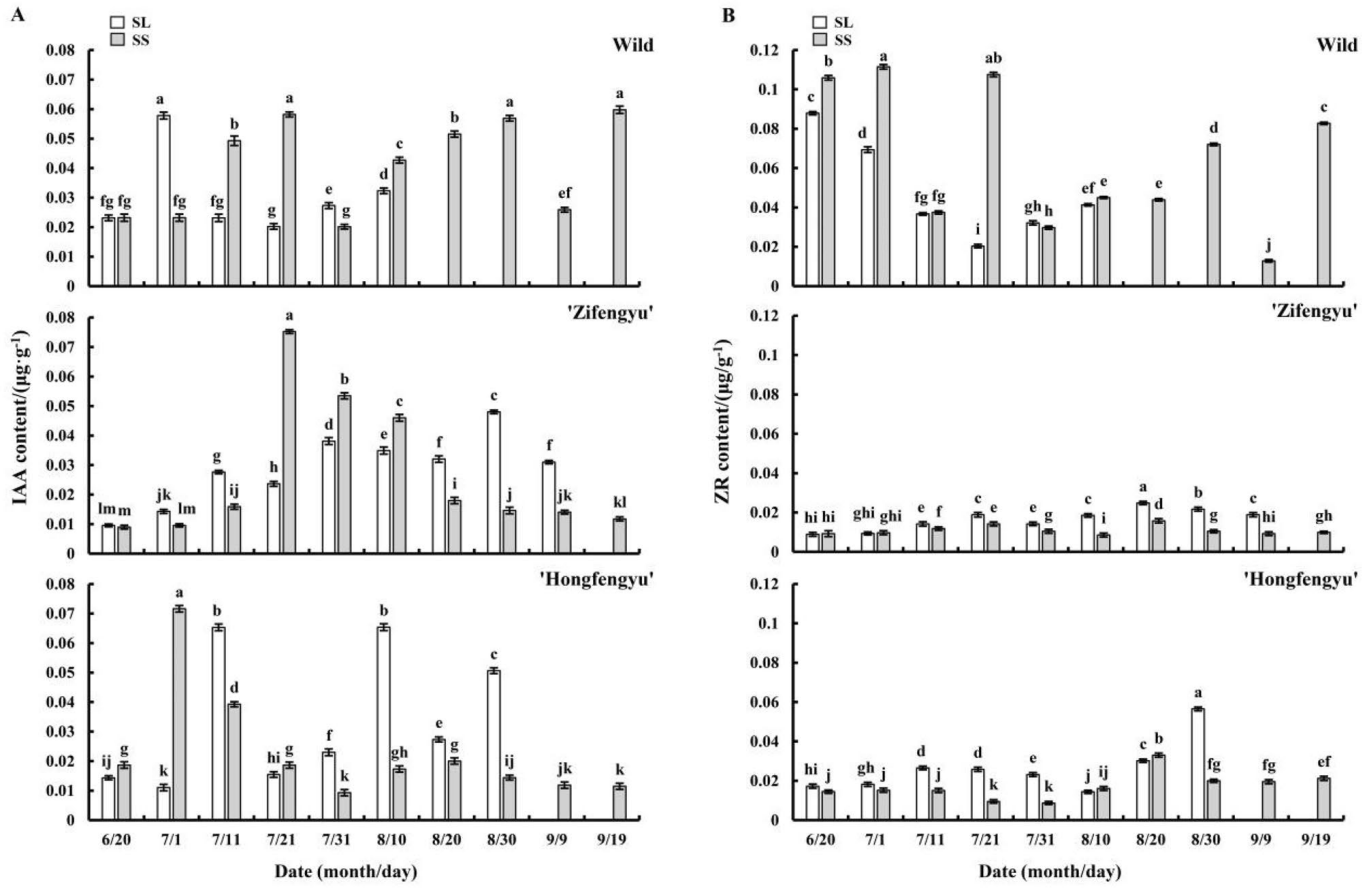
Changes in leaf auxin (IAA) and zeatin (ZR) contents under the SL and SS treatments. SL, strong light; SS, slight shading. Data are shown as mean ± standard error (*n* = 3). Different letters indicate significant differences among treatments according to Duncan’s test (*P* < 0.05).

As shown in Fig. 7A, from June 20 to September 19, variations the in IAA contents of ‘Zifengyu’ and ‘Hongfengyu’ under the SS treatment presented a unimodal trend, with peak values on July 21 and July 1, respectively. The IAA content of the wild species under the SS treatment showed a bipeak trend, with peaks on July 21 and August 30. The IAA content of the wild species under the SL treatment showed an increasing trend.

#### Changes in endogenous ZR content

As shown in Fig. 7B, the SS treatment reduced the leaf ZR content of ‘Zifengyu’ and ‘Hongfengyu’. From June 20 to September 19, the leaf ZR content of all tested materials showed a bimodal trend. The double peaks of ‘Zifengyu’ under the SS and SL treatments were consistent on July 21 and August 20. The double peak values of ‘Hongfengyu’ under the SS treatment appeared on July 11 and August 20, and those under the SL treatment appeared on July 11 and August 30. The double peak values of the wild species under the SS treatment appeared on July 1 and September 19, and those under the SL treatment appeared on June 20 and August 10.

#### Changes in endogenous GA_3_ content

**Figure 8:**
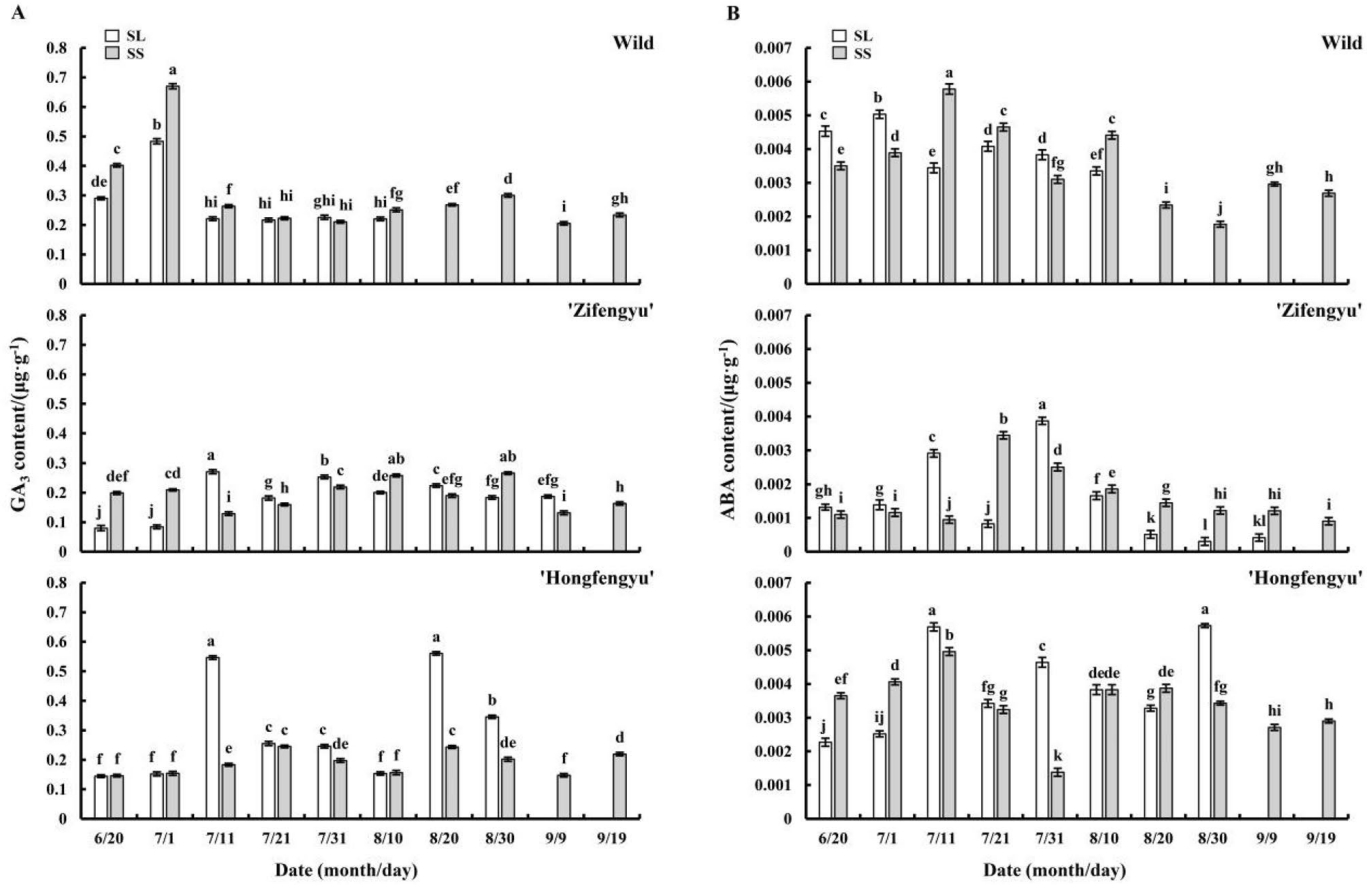
Changes in leaf gibberellin (GA_3_) and abscisic acid (ABA) contents under the SL and SS treatments. SL, strong light; SS, slight shading. Data are shown as mean ± standard error (*n* = 3). Different letters indicate significant differences among treatments according to Duncan’s test (*P* < 0.05).

As shown in Fig. 8A, the SS treatment increased the leaf GA_3_ content of all tested materials. From June 20 to September 19, the leaf GA_3_ content under the SS treatment showed a bipeak trend, with the peaks for ‘Zifengyu’ appearing on July 1 and August 10, those for ‘Hongfengyu’ appearing on July 21 and August 20, and those for the wild species appearing on July 1 and August 30. The GA_3_ content of ‘Zifengyu’ and ‘Hongfengyu’ under the SL treatment showed a bimodal trend. The peaks for ‘Zifengyu’ appeared on July 11 and July 31, and those of ‘Hongfengyu’ appeared on July 11 and August 20. The leaf GA_3_ content of the wild species under the SL treatment showed a unimodal trend, with a peak appearing on July 1.

#### Changes in endogenous ABA content

As shown in Fig. 8B, the SS treatment reduced the leaf ABA content of all tested materials. From June 20 to September 19, the ABA content of tested materials under the SS treatment showed a unipeak trend, with the peaks of ‘Zifengyu’, ‘Hongfengyu’, and the wild species appearing on July 21, July 11, and July 11, respectively. The leaf ABA content of ‘Zifengyu’ and ‘Hongfengyu’ under the SL treatment showed a bimodal trend. The peak values of ‘Zifengyu’ were observed on July 11 and July 31, and those of ‘Hongfengyu’ were observed on July 11 and August 30. The leaf ABA content of the wild species under the SL treatment showed a unimodal trend with a peak on July 11.

### Changes in leaf polyamine content under strong light and slight shading treatments

#### Changes in endogenous spermine content

**Figure 9:**
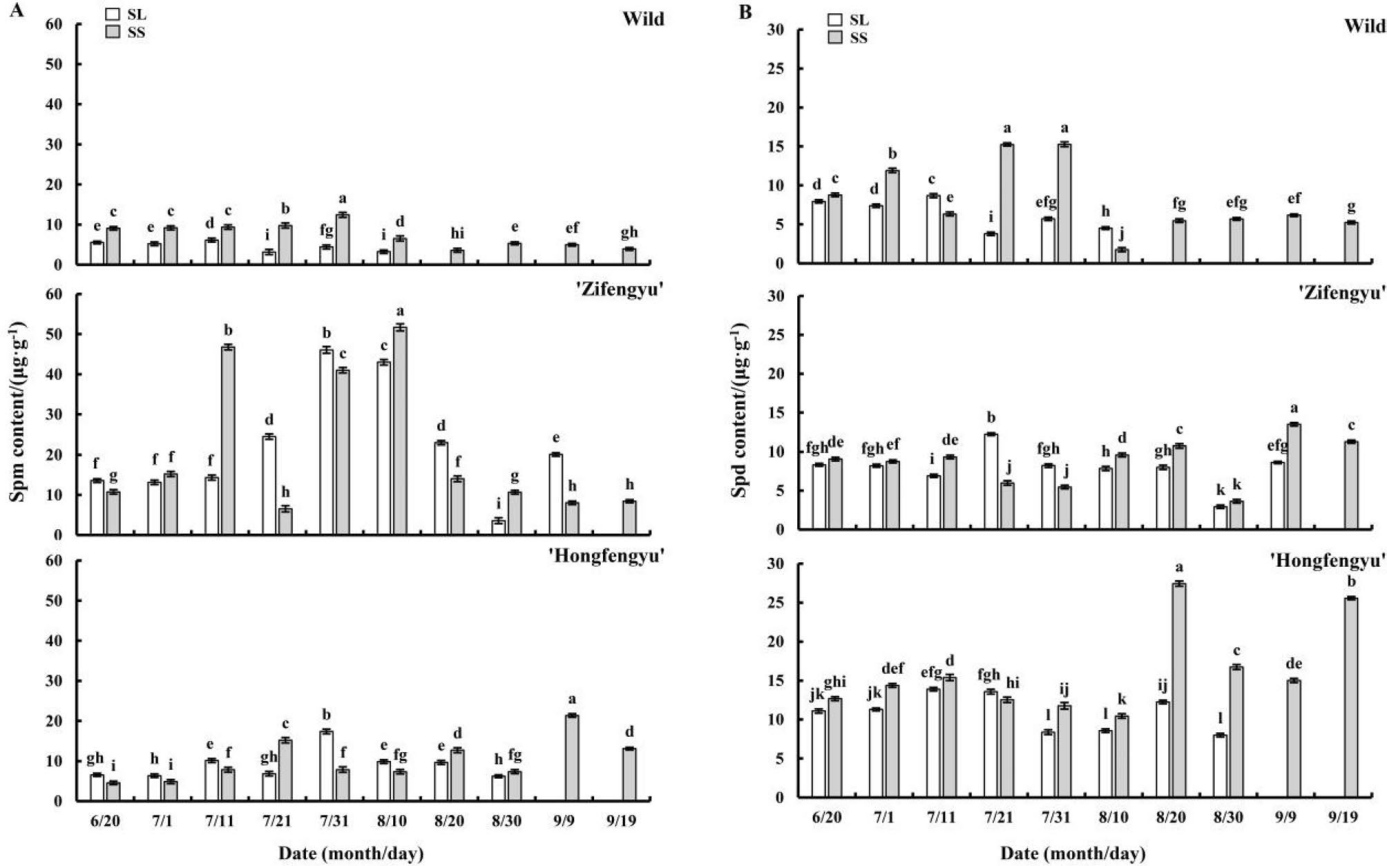
Changes in leaf spermine (Spm) and spermidine (Spd) contents under the SL and SS treatments. SL, strong light; SS, slight shading. Data are shown as mean ± standard error (*n* = 3). Different letters indicate significant differences among treatments according to Duncan’s test (*P* < 0.05).

As shown in Fig. 9A, the SS treatment increased the leaf Spm content of all tested materials. From June 20 to September 19, the leaf Spm content of ‘Zifengyu’ and the wild species under the SS treatment showed a unipeak trend with peaks on August 10 and July 31, respectively. The leaf Spm content of ‘Hongfengyu’ under the SS treatment showed an increasing trend. The leaf Spm content of ‘Zifengyu’, ‘Hongfengyu’, and the wild species under the SL treatment showed a unimodal trend with peaks of 7.31, 7.31, and 7.11, respectively.

#### Changes in endogenous spermidine content

As shown in Fig. 9B, the SS treatment increased the leaf Spd content of all tested materials. From June 20 to September 19, the leaf Spd content of ‘Zifengyu’ and ‘Hongfengyu’ under the SS treatment showed an increasing trend, whereas that of the wild species showed a unipeak trend and decreased to a stable level after July 31. The leaf Spd content of ‘Zifengyu’, ‘Hongfengyu’, and wild species under the SL treatment showed a unipeak trend with peaks on July 21, July 21, and July 11, respectively.

#### Changes in leaf putrescine content

**Figure 10:**
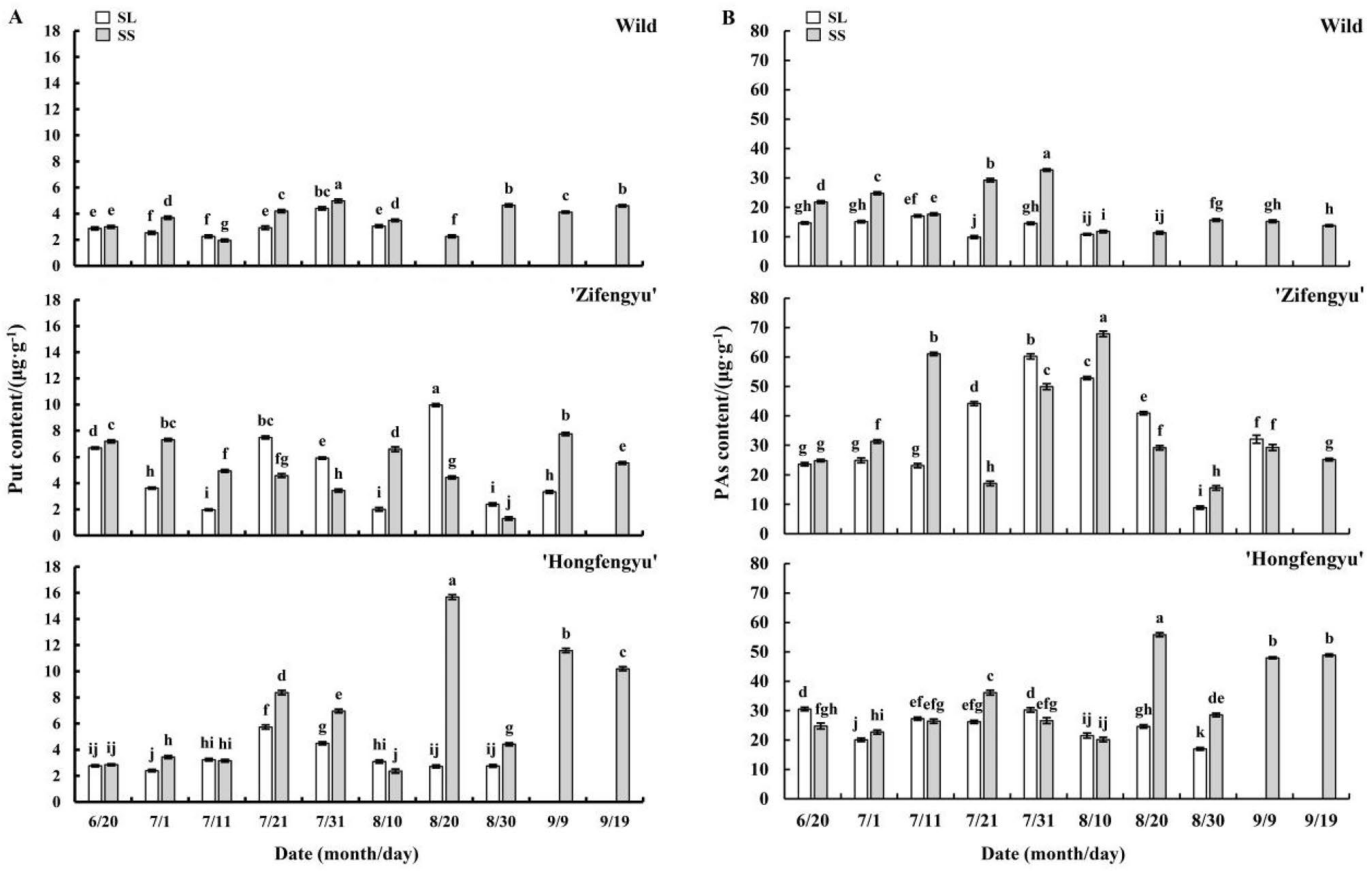
Changes in leaf putrescine (Put) contents under the SL and SS treatments. SL, strong light; SS, slight shading. Data are shown as mean ± standard error (*n* = 3). Different letters indicate significant differences among treatments according to Duncan’s test (*P* < 0.05).

As shown in Fig. 10A, the SS treatment increased the leaf Put content of all tested materials. From June 20 to September 19, variations in the leaf Put contents of all tested materials under the SS treatment showed a bipeak trend; ‘Zifengyu’ exhibited peak values on July 1 and September 9, ‘Hongfengyu’ exhibited peak values on July 21 and August 20, and the wild species exhibited peak values on July 31 and August 30. The leaf Put content of ‘Hongfengyu’ and the wild species under the SL treatment showed a unipeak trend, with peak values on July 21 and July 31, respectively. The leaf Put content of ‘Zifengyu’ under the SL treatment showed a bimodal trend with peaks on July 21 and August 20, respectively.

#### Changes in leaf polyamine content

As shown in Fig. 10B, the SS treatment significantly increased the leaf PA content of all tested materials. From June 20 to September 19, the leaf PA content of ‘Zifengyu’ and the wild species under the SS treatment showed a unipeak trend, with peaks on August 10 and July 31, respectively. The leaf PA content of ‘Hongfengyu’ under the SS treatment showed an increasing trend. The leaf PA content of ‘Zifengyu’, ‘Hongfengyu’, and the wild species under the SL treatment showed a unipeak trend, with peaks on July 31, July 31, and July 11, respectively.

## Discussion

Under high summer light intensity, plant leaves exhibit photo-inhibition and severe leaf scorching, reducing their photosynthetic capacity^21^. Shading can prevent summer leaf burning and improve vegetative growth^22^ Consistently, Qi^23^ found that shading reduced the quantum flux density of ambient light, ambient light intensity, and temperature, thus improving the microenvironment for herbaceous peony growth in summer. In our study, the slight shading treatment significantly reduced the PAR and Tl of the tested materials, in accordance with Qi’s (2018) findings. Furthermore, the leaf microstructure clearly reflects the response mechanism of plants to environmental change. Our results showed that the thickness of the leaves, mesophyll, palisade tissue, spongy tissue, and upper and lower epidermis of the tested materials under the summer strong light treatment was greater than that under the slight shading treatment, and the palisade tissue cells under the summer strong light treatment were more tightly arranged and orderly than those under the slight shading treatment. This indicated that herbaceous peony can block summer strong light by increasing leaf thickness and firmness. Similarly, Zhou et al.^24^ found that when rhododendron seedlings were shaded, the plant light saturation point, net photosynthetic rate, plant height, leaf area, and biomass accumulation significantly increased. Our results showed that slight shading could significantly delay leaf senescence in herbaceous peony, which was in accordance with Han et al.^25^ who studied the leaves of ‘Fengdan’. At the same time, we also found that ‘Zifengyu’ showed greater resistance to summer high-light intensity, and the wild species showed the worst resistance to summer high-light intensity.

Moderate shading can prevent excessive radiation from exceeding the light saturation point of photosynthesis to maintain high photosynthetic activity and reduce photo-inhibition^26^. In our study, the slight shading treatment during summer increased the leaf chlorophyll content of ‘Zifengyu’ and ‘Hongfengyu’ and increased the leaf net photosynthetic rate of all tested materials, similar to the results observed in tea plants by Chen et al.^27^. Soluble protein and proline are important osmoregulatory substances that regulate stomatal opening, photosynthesis, cell growth, and plant senescence^28^. Furthermore, the synthesis of many soluble proteins in the leaves is regulated by light^29^. In our study, the leaf soluble protein content of the tested materials under the summer strong light and slight shading conditions showed an increasing trend in the early stage of the experiment, with no significant difference between the two treatment conditions. In the late stage of the experiment, the leaf soluble protein content under the summer strong light treatment showed a decreasing or stable trend; however, the slight shading treatment showed an increasing or stable trend in the late stage of the experiment. The slight shading treatment increased the leaf soluble protein content, which enhanced the stress resistance of herbaceous peony. This result was consistent with that of Zhu^30^ who found that under shading, the soluble protein content in herbaceous peony leaves increased. Under the strong light treatment in summer, the leaf proline content of all tested materials showed an increasing trend, particularly in the wild species. However, under the slight shading treatment, the leaf proline content of all tested materials remained low, likely because of the high leaf temperature under the strong light conditions and the need to maintain a normal cell turgor pressure and improve the water retention ability. The variation range of the leaf proline content of ‘Hongfengyu’ and the wild species was significantly higher than that of ‘Zifengyu’; this was because different herbaceous peony cultivars have different responses to strong light stress.

Internal developmental signals and environmental factors induce hormones that regulate leaf senescence, and light affects the synthesis of endogenous hormones^31^. Zong et al.^32^ found that the endogenous hormone levels (ABA, IAA, ZR, and GA_3_) of *Leymus chinensis* increased under different shading conditions. Auxin affects cell growth and plant morphogenesis, and its exact role in leaf senescence is complex and controversial^33,34^. We found that the leaf IAA content exhibited a greater decrease in ‘Hongfengyu’ than in ‘Zifengyu’ under the slight shading treatment, and in the early stage, the increase in leaf IAA of the wild species was lower than that of ‘Zifengyu’ and remained stable in the late stage. This finding indicated that compared to ‘Zifengyu’, ‘Hongfengyu’ and the wild species were more effective in reducing the IAA content in the late stage of light shading, thereby significantly delaying or inhibiting further increases in IAA in the plant leaves. Gibberellin inhibits the degradation of plastids as well as the expression or transcription of aging genes^35^. Rather than directly regulating or acting on the aging mechanism, gibberellin antagonizes ABA to delay aging^6^. In the present study, the slight shading treatment increased the leaf GA_3_ content of all tested materials, thereby delaying the aging process. Cytokinin not only stimulates protein and nucleic acid synthesis but also enhances plant stress resistance; however, ABA has the opposite effects ^36^. Our results showed that under the slight shading treatment, the increase in the leaf zeatin content of ‘Hongfengyu’ and the wild species was greater than that of ‘Zifengyu’. Moreover, the decrease in the leaf ABA content of ‘Hongfengyu’ and the wild species was greater than that of ‘Zifengyu’, indicating that ‘Hongfengyu’ and the wild species were more effective than ‘Zifengyu’ in increasing the zein content and reducing the ABA content under the slight shading treatment to enhance their resistance and inhibit the aging process.

Polyamine levels are influenced by developmental responses such as maturation and senescence^37,38^. Furthermore, polyamines delay plant senescence by inhibiting chlorophyll and protein degradation^39,40^. Many studies have shown that the application of exogenous polyamines delays plant senescence^13,41,42^. In our study, shading increased the leaf Put, Spd, Spm, and PA contents in the two herbaceous peony cultivars and the wild species and delayed leaf senescence. Under the slight shading treatment, the contents of leaf endogenous Put, Spd, Spm, and PAs of ‘Hongfengyu’ and the wild species increased more than those of ‘Zifengyu’. Compared with ‘Zifengyu’, ‘Hongfengyu’ and the wild species showed increased endogenous polyamine contents under the slight shading treatment.

## Conclusions

Slight shading in summer increased the contents of IAA, ZR, GA_3_, Spm, Spd, Put, chlorophyll, and net photosynthetic rate and decreased the leaf ABA, thereby delaying leaf senescence. The tolerance of peony cultivars to summer high-light intensity stress ranked in the following order: ‘Zifengyu’ > ‘Hongfengyu’ > wild species. The increase in the GA_3_, ZR, Put, Spd, Spm, and PA contents in the leaves of ‘Hongfengyu’ and the wild species was greater than that in ‘Zifengyu’ leaves. The increase in the IAA content in ‘Hongfengyu’ was lower than that in ‘Zifengyu’, and the decrease in the ABA content in ‘Hongfengyu’ was greater than that in ‘Zifengyu’. These results indicated that under the SS treatment, ‘Hongfengyu’ and the wild species were able to delay the senescence of their leaves more effectively than ‘Zifengyu’. This study provides a theoretical basis for using summer shading facilities and growth regulators to delay leaf senescence of herbaceous peony. At the same time, this study provides a theoretical reference for breeding high-intensity light-resistant herbaceous varieties, realizing high-efficiency and high-yield cultivation of herbaceous peony, and for their popularization and utilization.

## Data Availability

The datasets used and analysed during the current study available from the corresponding author on reasonable request.
